# Dimethyl fumarate improves cognitive impairment and neuroinflammation in mice with Alzheimer’s disease

**DOI:** 10.1186/s12974-024-03046-2

**Published:** 2024-02-21

**Authors:** Ting Wang, Akira Sobue, Seiji Watanabe, Okiru Komine, Takaomi C. Saido, Takashi Saito, Koji Yamanaka

**Affiliations:** 1 Department of Neuroscience and Pathobiology, Research Institute of Environmental Medicine, Nagoya University, Aichi, 464-8601 Japan; 2 Department of Neuroscience and Pathobiology, Nagoya University Graduate School of Medicine, Aichi, Japan; 3 Medical Interactive Research and Academia Industry Collaboration Center, Research Institute of Environmental Medicine, Nagoya University, Aichi, Japan; 4 Laboratory for Proteolytic Neuroscience, RIKEN Center for Brain Science, Saitama, Japan; 5 Department of Neurocognitive Science, Institute of Brain Science, Nagoya City University Graduate School of Medical Sciences, Aichi, Japan; 6 Institute for Glyco-Core Research (iGCORE), Nagoya University, Aichi, Japan; 7 Center for One Medicine Innovative Translational Research (COMIT), Nagoya University, Nagoya, Aichi Japan

**Keywords:** Alzheimer’s disease, Astrocytes, Neuroinflammation, STAT3, Nrf2, Dimethyl fumarate

## Abstract

**Background:**

Neuroinflammation substantially contributes to the pathology of Alzheimer’s disease (AD), the most common form of dementia. Studies have reported that nuclear factor erythroid 2-related factor 2 (Nrf2) attenuates neuroinflammation in the mouse models of neurodegenerative diseases, however, the detailed mechanism remains unclear.

**Methods:**

The effects of dimethyl fumarate (DMF), a clinically used drug to activate the Nrf2 pathway, on neuroinflammation were analyzed in primary astrocytes and *App*^NL−G−F^ (*App*-KI) mice. The cognitive function and behavior of DMF-administrated *App*-KI mice were evaluated. For the gene expression analysis, microglia and astrocytes were directly isolated from the mouse cerebral cortex by magnetic-activated cell sorting, followed by quantitative PCR.

**Results:**

DMF treatment activated some Nrf2 target genes and inhibited the expression of proinflammatory markers in primary astrocytes. Moreover, chronic oral administration of DMF attenuated neuroinflammation, particularly in astrocytes, and reversed cognitive dysfunction presumably by activating the Nrf2-dependent pathway in *App*-KI mice. Furthermore, DMF administration inhibited the expression of STAT3/C3 and C3 receptor in astrocytes and microglia isolated from *App*-KI mice, respectively, suggesting that the astrocyte–microglia crosstalk is involved in neuroinflammation in mice with AD.

**Conclusion:**

The activation of astrocytic Nrf2 signaling confers neuroprotection in mice with AD by controlling neuroinflammation, particularly by regulating astrocytic C3-STAT3 signaling. Furthermore, our study has implications for the repositioning of DMF as a drug for AD treatment.

**Supplementary Information:**

The online version contains supplementary material available at 10.1186/s12974-024-03046-2.

## Introduction

Alzheimer’s disease (AD) is the most common cause of dementia affecting elderly individuals. Patients with AD typically show a progressive decline in memory, language ability, and executive functions. Accumulation of amyloid-β and phosphorylated tau is a well-known neuropathological hallmark of AD [1] On the other hand, neuroinflammation, defined as an inflammatory response of the central nervous system (CNS), is mediated by activation of the innate immune system of the brain in response to various inflammatory challenges, including protein misfolding and aggregation, which are often found in neurodegenerative diseases, including AD [2–4]. Because the pathophysiology of the disease is only partially understood and the available therapies are limited, novel therapeutic options based on an understanding of AD pathomechanism are awaited [1].

Nuclear factor-erythroid 2-related factor 2 (Nrf2), a transcription factor encoded by *NFE2L2*, is a member of a small family of basic leucine zipper (bZIP) proteins. Nrf2 is a master regulator of cellular stress responses by inducing the expression of cytoprotective genes that contain the antioxidant response element (ARE) in their promoter region [5]. Nrf2 target genes are involved in inflammation, detoxification reactions, redox balance and energy metabolism, and proteostasis, all of which have been implicated in the pathomechanism of neurodegenerative diseases [5, 6]. Accumulating evidence suggests that Nrf2 is expressed at low levels in the brain tissue of patients with AD [7, 8] and that Nrf2 activation can reduce several pathological features of AD in mice [9]. Conversely, genetic ablation of Nrf2 exacerbates neuroinflammation, neuropathology, and cognitive dysfunction in AD mouse models [10–13]. Anti-inflammation is also a prominent feature of Nrf2 activation, which causes transcriptional upregulation of Nrf2 target genes (e.g., *HMOX1*, *NQO1*, and *GCLM*) and downregulation of the genes that encode major proinflammatory cytokines [14, 15]. Nrf2 is particularly enriched in glial cells [16, 17], which substantially contribute to antioxidant and anti-inflammatory defenses, many of which are regulated by Nrf2 [18]. Several studies have shown that Nrf2 activation can inhibit microglial and/or astrocyte activation in AD rodent models [9] and provide protection against microglia-induced neuronal death [19]. Furthermore, Nrf2 expression in astrocytes conferred neuroprotection in experimental mouse models of several neurodegenerative diseases [20, 21]. All these findings suggest that increasing Nrf2 activity in CNS cells, particularly glial cells, is a potential option for AD treatment.

Nrf2 activity is tightly regulated at the level of *NFE2L2* and by its protein stability [22]. Interaction with the E3 ligase adapter Kelch-like ECH-associated protein 1 (KEAP1), which controls Nrf2 protein stability under basal redox conditions, is the major Nrf2 regulatory mechanism [18]. Dimethyl fumarate (DMF), an approved drug for multiple sclerosis, can disrupt the KEAP1–Nrf2 interaction by binding to cysteine residues within KEAP1, allowing Nrf2 translocation to the nucleus and Nrf2 transactivation of target genes by binding to their AREs [23]. Although DMF has been shown to be beneficial in models of neurodegenerative diseases, such as Parkinson’s disease and Huntington’s disease [24, 25], the results were not consistent in DMF-administered mouse models of AD [10, 26]. Therefore, whether DMF-mediated activation of the Nrf2 pathway confers neuroprotection in mice with AD by controlling neuroinflammation and its detailed molecular basis remains to be determined.

In this study, whether oral administration of DMF controls neuroinflammation and ameliorates cognitive decline in mice with AD was examined. Furthermore, the detailed gene expression profile of isolated glial cells in DMF-administered mice with AD was analyzed.

## Materials and methods

### Animals

Heterozygous *App*^+*/NL−G−F*^ mice (C57BL/6-App < tm3(NL-G-F)Tcs >), carrying the *App* gene with humanized Aβ sequence (G676R, F681Y, and R684H), Swedish (KM670/671NL), Beyreuther/Iberian (I716F), and Arctic (E693G) mutations, were previously established by a knock-in strategy [27]. Homozygous *App*^*NL−G−F/NL−G−F*^ mice were obtained by crossbreeding. Genotyping of mice was performed as previously described Age-matched wild‐type control mice (C57BL/6J) were purchased from CLEA Japan (Tokyo, Japan). Nrf2 knockout mice (B6.129P2-Nfe2l2 < tm1Mym > /MymRbrc, RBRC01390) were obtained from the RIKEN BioResource Research Center (Tsukuba, Japan) with the kind permission of Dr. Masayuki Yamamoto (Tohoku University) [28] All mice were maintained under a standard specific pathogen-free environment (12 h light–dark cycle; 23 ± 1 ºC; 50 ± 5% humidity) with free access to food and water throughout the experiments. The animals were treated in compliance with the guidelines established by the Institutional Animal Care and Use Committee of Nagoya University.

### Isolation of microglia and astrocytes from the mouse brain

Microglia and astrocytes were isolated from the cerebral cortices of mice using magnetic-activated cell sorting (MACS) as described previously [29]. In brief, after mice were transcardially perfused with phosphate-buffered saline (PBS) under deep anesthesia, the cerebral cortex was dissociated at 37 °C for 15 min using the Neural Tissue Dissociation Kit-Postnatal Neurons (Miltenyi Biotec, Bergisch-Gladbach, Germany) with a gentle MACS Dissociator (Miltenyi Biotec). To isolate microglia, we removed myelin debris using Myelin Removal Beads II (Miltenyi Biotec) and incubated purified cells with anti-CD16/CD32 antibodies (Thermo-Fisher Scientific, Waltham, MA, USA) for blocking Fc receptors, followed by incubation with anti-CD11b MicroBeads (Miltenyi Biotec). By MACS, CD11b-positive microglia were isolated through an LS column (Miltenyi Biotec). To isolate astrocytes, astrocyte-containing, CD11b-negative flow-through cells were incubated with anti-ACSA2 MicroBeads (Miltenyi Biotec), and then subjected to MACS through the LS column.

### Chronic oral administration of dimethyl fumarate

DMF (Sigma, CA, USA) was dissolved in corn oil and administered by oral gavage (300 mg/kg, p.o) [30]. We administered DMF orally three times per week for 1 month (short-term administration) and 5 months (long-term administration). To minimize bias because of possible undetected changes in environmental conditions, Veh- or DMF-administered wild-type (WT)/App mice were always examined in pairs; both recordings were performed on the same day. Therefore, the experimenter was not blinded to the genotype/treatment throughout the experimental procedures. No exclusion criteria were predetermined, and no animals were excluded. The sample size for each experiment was determined based on previous studies with the relevant type of experiment [29, 31].

### Behavioral experiments

The novel object recognition test was performed as described previously [31] with minor modifications. The mice were habituated to an open box (30 × 30 × 35 cm) individually for 3 days. During the training, two novel objects were placed in an open field. Under moderately lit conditions (12 lx), mice were allowed to explore for 10 min and the time spent exploring each object was recorded. During the test sessions, one of the familiar objects used in the training session was replaced by a novel object. The mice were placed back into the same box 24 h after the training session and allowed to explore freely for 5 min. The preference index in the test session, which is the ratio of the duration of time spent exploring the novel object to the total time spent exploring both objects, was used to evaluate cognitive function.

An open field test was performed for measuring exploratory behavior and anxiety in a novel environment according to the manufacturer’s protocol (O’Hara & Co., Ltd., Tokyo, Japan). The mice were placed in the center of an empty open field (40 × 40 × 30 cm) and allowed to explore the environment for 20 min without any external disturbances. TimeOFCR1 software (O’Hara & Co., Ltd.) was used to measure the movement patterns, time spent in the center and margin, velocity, and distance traveled; these measurements were used as indicators of anxiety, agility, and exploration.

### Quantification of mRNA levels using real-time PCR

According to the manufacturer’s instructions, we used the RNeasy Micro Kit (Qiagen) to extract total RNA from cultured astrocytes or MACS-isolated microglia and astrocytes. Complementary DNA (cDNA) from MACS-isolated cells was generated by reverse transcription of total RNA (2.5 or 5 ng) using the PrimeScript™ RT reagent Kit (Perfect Real Time) (TaKaRa Bio, Kusatsu, Japan), and 1/50 of the yield was amplified using the SYBR Premix Ex Taq II (Tli RNaseH Plus) (TaKaRa Bio) and the Thermal Cycler Dice Real Time System II or III (TaKaRa Bio). The PCR protocol was as follows: 1 cycle at 95 °C for 30 s; 40 cycles at 95 °C for 5 s and 60 °C for 30 s; and a dissociation stage at 95 °C for 15 s, 60 °C for 30 s, and 95 °C for 15 s. *Actb* was used for normalization. The primers used for real-time RT-PCR are summarized in Additional file 1: Table S1.

### Immunofluorescence analysis

Immunofluorescence analysis was performed as described previously [29]. In brief, mice were deeply anesthetized and perfused intracardially with PBS and 4% paraformaldehyde in PBS. The brains were dissected, postfixed with the same fixative, and cryoprotected with 30% sucrose containing PBS. Twenty-micrometer-thick coronal brain sections were fixed with 4% paraformaldehyde in PBS for 5 min, and then we performed antigen retrieval with HistoVT One (Nakarai, Kyoto, JAPAN) 70 °C for 20 min. After incubation in blocking solution (5% goat or donkey serum/PBS) for 1 h, sections were incubated with a combination of the following antibodies: rabbit anti-Iba-1 (#019–19741, 1:500; FUJIFILM Wako Osaka, Japan), goat anti-AIF-1/Iba1 (#NB100-1028, 1:250; Novus Biologicals, CO, USA), mouse anti-Aβ (#10326, 1:200; IBL, Nagoya, Japan), mouse anti-GFAP (#G3893, 1:250, Sigma), rat anti-C3 (#sc58926, 1:200; Santa Cruz Biotechnology, Inc., CA, USA), rabbit anti-BACE1 (#5606, 1:100, Cell Signaling, Inc., CA, USA), and rabbit anti-p-STAT3 (Tyr705) (#9145, 1:2000, Cell Signaling Technology, Inc.) at 4 °C overnight. After washing with PBS, the sections were incubated with fluorescent-conjugated anti-rabbit, anti-mouse, anti-goat, or anti-rat IgG (1:1000; Thermo Fisher Scientific) and 4ʹ,6ʹ-diamidino-2-phenylindole (DAPI) (1:2000) at room temperature for 1 h. After washing, the sections were mounted on slides with Fluoromount/Plus™ (Diagnostic BioSystems, Pleasanton, CA, USA) and analyzed using a confocal microscope (LSM700, Carl Zeiss, Oberkochen, Germany). Microscopic images were quantitatively analyzed using ImageJ (National Institutes of Health).

### Protein extraction and immunoblotting

The half brain hemisphere of mice was homogenized in RIPA buffer (tris-buffered saline (TBS) with 1% NP-40, 1% sodium deoxycholic acid, 0.1% sodium dodecyl sulfate (SDS), complete protease inhibitor cocktails (Roche), and PhosSTOP phosphatase inhibitor cocktails (Roche)) and centrifuged at 20,000 × g for 30 min. The resulting supernatant was used as the RIPA–soluble fraction. The remaining brain hemisphere was used for Aβ extraction as described elsewhere [32]. In brief, mouse brains were homogenized in 5 × volumes of TBS (50 mM Tris–HCl pH7.6, 150 mM NaCl, complete protease inhibitor cocktail, and PhosSTOP phosphatase inhibitor cocktail) with 25 strokes using a potter homogenizer and centrifuged at 200,000 × g at 4 °C for 20 min. The resulting supernatant was collected as a TBS-soluble fraction. After the addition of the same amount of 2% Triton X-100/TBS, the pellet was homogenized on ice and centrifuged at 200,000 × g at 4 °C for 20 min. The resulting supernatant was collected as the Triton X fraction. Then, the same amount of 2% SDS containing TBS was added to the pellet. After homogenization at room temperature, the pellet was incubated at 37 °C for 2 h and centrifuged at 200,000 × g at 20 °C for 20 min. The resulting supernatant was collected as the SDS fraction. Finally, the pellet was sonicated with 500 μL of 70% formic acid (WAKO). The samples were centrifuged at 200,000 × g at 4 °C for 20 min, and the resulting supernatant was evaporated for 2 h. The pellet was dissolved in the same volume of dimethyl sulfoxide (DMSO) as the brain weight and stored at − 80 °C until use. To determine the protein concentration, the BCA assay was used. Equal amounts of total protein were separated using SDS–polyacrylamide gel electrophoresis (SDS-PAGE) and transferred to a polyvinylidene difluoride membrane (Immobilon-P; Merck Millipore, Billerica, MA, USA). The membrane was incubated with a blocking buffer (50 mM Tris–HCl (pH7.4), 150 mM NaCl, 0.05% (v/v) Tween-20, and 2% (w/v) bovine serum albumin (FUJIFILM Wako), followed by incubation with mouse anti-Aβ (#10323, 1:200; IBL, Nagoya, Japan), mouse anti-ACTB (#a5441, 1:5000; Sigma), rabbit anti-human APP (C) (#18961, 1:5000; IBL, Nagoya, Japan), mouse anti-GFAP (#G3893, 1:250, Sigma), rabbit anti-p-STAT3(Tyr705) (#9145, 1:2000, Cell Signaling Technology Inc., CA, USA), rabbit anti-Nrf2 (#137550, 1:1000, abcam, Cambridge, UK), rabbit anti-SOD1 (#ab_10616253, Enzo Life Science, Farmingdale, NY, USA), or rabbit anti-fibrillarin (C13C3) (#2639, 1:5000, Cell Signaling, Inc, CA, USA) antibodies diluted in the blocking buffer at 4 °C for at least 6 h. The membrane was further incubated with the corresponding horseradish peroxidase-conjugated secondary antibodies and visualized using Immobilon Crescendo Western HRP substrate (Merck Millipore). Images were obtained using LAS-4000 mini (Cytiva, Shinjuku, Tokyo, Japan) with the equipped software (Multi-Gauge; Cytiva). Silver staining of SDS–polyacrylamide gel was performed according to the manufacturer’s instructions (FUJIFILM Wako).

### Cell culture and drug treatment

Mouse primary astrocytes were prepared as described previously with minor modifications [33]. Briefly, primary astrocytes were prepared from the brains of newborn (postnatal days 0–3) C57/BL6J or Nrf2 knockout mice. The brain was isolated and carefully stripped off the meninges in PBS. The tissue was minced into small pieces, digested by incubation with 0.25% trypsin and 0.01% DNase at 37 ºC for 10 min, and mechanically dissociated by gentle pipetting. Debris was removed by passing the samples through a 70 μm pore cell strainer. The resulting cells were plated onto a poly-L-lysine (Sigma)-coated flask (25 cm^2^), and cultured in glial medium (Dulbecco’s Modified Eagle’s Medium (DMEM; Sigma) containing 10% (v/v) fetal bovine serum (Gibco, Gaithersburg, MD, USA). The culture medium was replenished after 24 h and incubated for 7 days. The astrocyte layer was detached using 0.25% trypsin EDTA (FUJIFILM Wako) and replated on culture plates. After replating twice, the primary astrocytes were used in all experiments. We confirmed that more than 95% of the cells were ALDH1L1-positive and Iba1-negative by immunostaining (ALDH1L1 (+): 98%, IBA1 (+): 0% and ALDH1L1 (−) / IBA1 (−): 2%). Primary astrocytes and the human glioma cell line T98G (RRID: CVCL_0556) were cultured in glial medium at 37 °C, 5% CO_2_ in air, and 95% humidity. Primary astrocytes were pretreated with 35 μM DMF or DMSO (vehicle; Veh) for 1 h followed by treatment with TNFα (30 ng/mL; Peprotech, Hartford, CT, USA), IL-1α (3 ng/mL; Peprotech, Hartford, CT, USA), and C1q (400 ng/mL; Hycult Biotech, PB Uden, The Netherlands) for 24 h as indicated [34]. The T98G cell line was treated with DMF (0 and 35 μM) for 6 h [35]. Treated cells were analyzed using real-time PCR or immunoblotting.

### Immunocytochemistry

Immunostaining was performed as previously described [31] with minor modifications. Astrocytes were seeded at a density of 7.0 × 10^4^ cells/well on LabTek II 4-well chamber slides (Thermo Fisher Scientific) coated with poly-D-Lysine (Sigma). The cells were fixed with 4% (w/v) paraformaldehyde for 20 min at room temperature, permeabilized with PBS containing 0.5% (v/v) Triton X-100 for 30 min., incubated in a blocking buffer (PBS containing 0.3% (v/v) Triton X-100 and 5% goat/donkey serum) for 1 h, and incubated with rat anti-C3 (#sc58926, 1:200; Santa Cruz Biotechnology, Inc, CA, USA), mouse anti-GFAP antibody (#G3893, 1:5000, Sigma) and rabbit anti-SOCS3 antibody (#ab16030,1:500, abcam), or mouse anti-ALDH1L1 antibody (sc100497, 1:50, Santa Cruz) diluted in blocking buffer at 4 °C for overnight. After washing three times with PBS containing 0.3% (v/v) Triton X-100, the slides were further incubated with Alexa-conjugated secondary antibodies and 0.5 μg/mL DAPI diluted in the blocking buffer for 60 min at room temperature, followed by embedding with Fluoromount/Plus (Diagnostic BioSystems). Confocal images were obtained using a confocal laser scanning microscopy (LSM-700; Carl Zeiss AG, Oberkochen, Baden-Württemberg, Germany) and analyzed using the equipped software (ZEN; Carl Zeiss AG). Microscopic images were quantitatively analyzed using ImageJ (National Institutes of Health).

### Subcellular fractionation

Subcellular fractionation was performed as previously described [36]. In brief, the cells were gently homogenized (Potter homogenizer, Wheaton) in isotonic buffer (10 mM HEPES, 250 mM sucrose, pH 7.4) supplemented with complete protease inhibitor and centrifuged at 600 × g at 4 °C for 5 min. The pellet was collected as P1. The supernatant was further centrifuged at 10,000 × g for 30 min at 4 °C. The supernatant was collected as the cytoplasmic fraction. The P1 pellet was resuspended in ice-cold hypotonic buffer (10 mM HEPES, 10 mM KCl, 1 mM MgCl_2_, 0.5 mM dithiothreitol (DTT), pH 7.4) and centrifuged at 600 × g at 4 °C for 5 min. The pellet was resuspended in ice-cold hypertonic buffer (10 mM HEPES, 400 mM NaCl, 1 mM MgCl_2_, 0.2 mM EGTA, 30% glycerol, 0.5 mM DTT, pH 7.4) and agitated at 4 °C for 30 min. After centrifugation at 18,000 × g at 4 °C for 30 min, the supernatant was collected as a nuclear fraction. The protein concentration of each sample was determined using a Bradford protein assay kit (Bio-Rad, Richmond, CA, USA).

### Statistical analysis

For behavioral experiments and quantitative PCR, all data are expressed as means ± SEM. The normality of the data was not assessed before the statistical comparisons. We opted to use parametric statistics for consistency across experiments and provided evidence that analysis of variance (ANOVA) is robust to slight non-normality [37, 38]. No test for outliers was performed. One-way or two-way ANOVA with or without repeated measures was used, followed by Tukey’s test when F ratios were significant (*p* < 0.05). Significant differences between the two groups were determined using the Student’s* t*-test. Detailed information on the statistical analysis is summarized in Additional file 2: Table S2.

## Results

### Dimethyl fumarate (DMF) pretreatment suppresses the proinflammatory astrocyte activation by activating the Nrf2 pathway

**Fig. 1:**
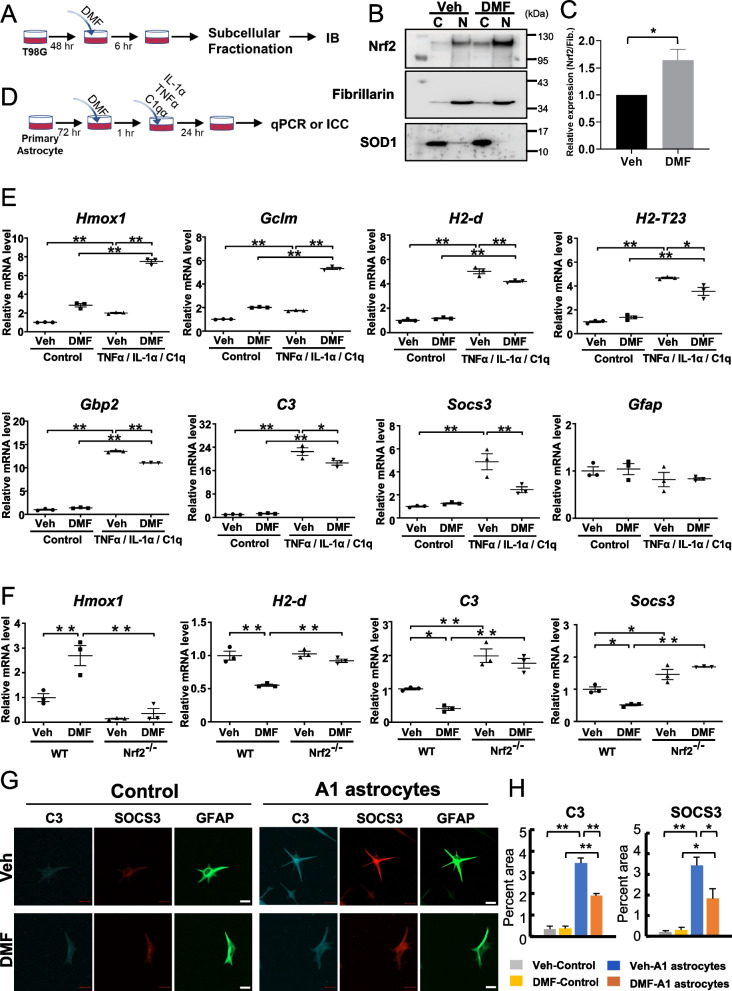
Dimethyl Fumarate (DMF) activates the Nrf2 pathway and inhibits the activation of proinflammatory markers in T98G glioma cells and primary astrocytes. **A** A schematic protocol for DMF treatment in the T98G glioma cell line. **B** Representative immunoblots for the lysates of T98G cells treated with or without DMF. DMF treatment activates the Nrf2 pathway in the T98G cell line. Cytosolic (Cyto) and nuclear (Nuc) fractions were subjected to immunoblotting using an anti-Nrf2 antibody. Fractionation was validated by immunoblotting for the fraction-specific marker proteins fibrillarin (nuclear marker) and SOD1 (cytosolic marker). Cytosolic and nuclear fractions were loaded with 10 µg of the protein per well. **C** Quantification of immunoblotting in B. Relative levels of nuclear Nrf2 normalized by the nuclear marker fibrillarin were quantified from three independent experiments and are expressed as means ± standard error of the mean (SEM) with *p*-values (n = 3, each group). *p < 0.05 (Student’s t-test). **D** Schematic protocol for DMF treatment in primary cultured A1 astrocytes. **E** Expression levels of mRNAs in DMF-treated primary astrocytes as determined by quantitative PCR. Relative expression levels for *Nfe2l2* downstream genes, such as *Hmox1* and *Gclm*; A1-astrocyte markers, such as *H2d*, *H2t23,* and *Gbp2*; and inflammatory and glial markers, such as *C3*, *Socs3,* and *Gfap,* are plotted as means ± SEM (Veh-Control: n = 3, DMF-Control: n = 3, Veh-A1 (TNFα/IL-1α/C1q): n = 3, and DMF-A1 (TNFα/IL-1α/C1q): n = 3). *p < 0.05 and **p < 0.01 (two-way ANOVA). **F** Expression levels of mRNAs in A1-induced primary astrocytes derived from WT and Nrf2^−/−^ mice determined by quantitative PCR. WT and Nrf2^−/−^ astrocytes were preincubated with TNFα/IL-1α/C1q in the presence or absence of DMF, as shown in **D**. Relative expression levels for *Hmox1*, *H2-d*, *C3*, and *Socs3* are plotted as means ± SEM (n = 3, each group). *p < 0.05 and **p < 0.01 (two-way ANOVA). **G** Representative immunofluorescent images demonstrating the expression of C3 (Blue), SOCS3 (Red), and GFAP (Green) in Veh-treated control or A1 astrocytes (upper panels, **G**) and DMF-treated control or A1 astrocytes (lower panels, **G**). Scale bars: 20 µm. **H** Quantification of immunofluorescence images in **G**. Percentage of the area immunopositive for C3 and SOCS3 in Veh- or DMF-treated control and A1 astrocytes. Values are represented as means ± SEM (n = 3, each group). *p < 0.05 and **p < 0.01 (two-way ANOVA)

Although previous studies have demonstrated anti-inflammatory effects of DMF in astrocytes via the Nrf2 pathway [39, 40], detailed molecular changes in glial cells have not been determined. To confirm whether DMF can activate the Nrf2 pathway in glial cells, we treated the astroglioma cell line T98G with DMF. Nrf2 activation was evaluated by the subcellular localization of Nrf2 using immunoblots (Fig. 1A). DMF treatment induced the nuclear translocation of Nrf2 in T98G cells, indicating that DMF can activate Nrf2 (Fig. 1B and C). A1 astrocytes, induced by the sets of cytokines in vitro, play a detrimental role in neuroinflammation in various neurological diseases [34]. To investigate the effects of Nrf2 activation on A1 astrocytes, we pretreated primary astrocytes with DMF and then administered IL-1α, TNFα, and C1q to induce A1 astrocytes (Fig. 1D). To determine whether DMF activated the Nrf2 pathway, we measured the expression of Nrf2 and its downstream genes (i.e., *Hmox1* and *Gclm*) using qPCR. The expression levels of *Nfe2l2* and its downstream genes were significantly upregulated in A1 astrocytes by DMF pretreatment (Fig. 1E and Additional file 3: Fig. S1A), which is consistent with the previous report [40]. To examine the role of Nrf2 in the induction of A1 astrocytes, we measured the expression levels of the A1 astrocyte markers *H2d*, *H2t23,* and *Gbp2*. As shown in Fig. 1E, the expression of *H2d*, *H2t23,* and *Gbp2* was upregulated in A1 astrocytes and was significantly suppressed by DMF. Furthermore, we found that the expression of *C3* and *Socs3* in A1 astrocytes was suppressed by DMF (Fig. 1E). No significant difference in the expression level of *Gfap* was observed between the groups. To confirm whether DMF suppressed the expression of proinflammatory genes in astrocytes through the Nrf2 pathway, we performed an experiment using primary astrocytes derived from Nrf2-deficient mice. In Nrf2-deficient astrocytes, the expression of the A1 astrocyte markers *H2-d* and *H2t23* and that of *C3* and *Socs3* were unaffected by DMF treatment (Fig. 1F and Additional file 3: Fig. S1B). The protein levels of C3 and SOCS3 were reduced in DMF-treated A1 astrocytes, as revealed by immunofluorescence analysis (Fig. 1G and H). These results suggest that DMF suppresses the proinflammatory phenotype in primary astrocytes, likely through Nrf2 activation.

### Short-term DMF administration regulates Nrf2-related genes in the astrocytes of *App*-KI mice at an early stage of the disease

**Fig. 2:**
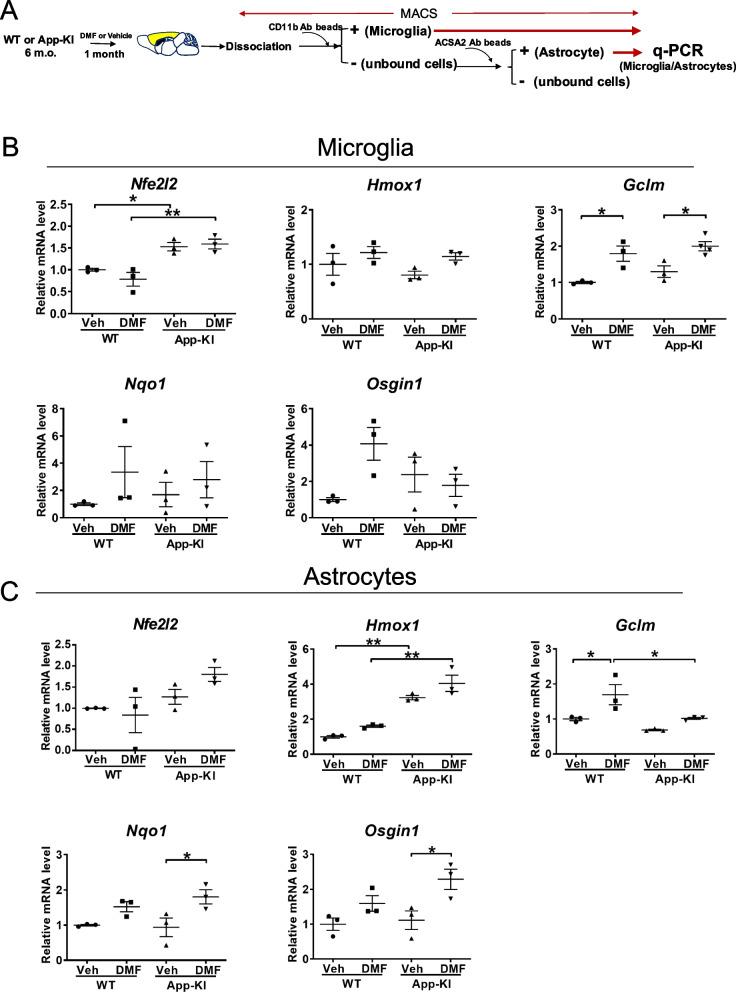
Short-term oral DMF administration affects the expression of neuroinflammatory molecules in glial cells of *App*-KI mice. **A** Schematic protocol for the oral administration of DMF and isolation of microglia and astrocytes using MACS. Six-month-old WT or *App*-KI mice were orally administered with DMF or vehicle control for 1 month. **B**, **C** mRNA expression levels in MACS-isolated microglia (**B**) and astrocytes (**C**) from DMF- or vehicle-administered WT and *App*-KI mice determined by quantitative PCR. (n = 3, each group). Relative expression levels are plotted as means ± SEM. *p < 0.05, **p < 0.01 (two-way ANOVA)

To identify the glial cell type that responds to DMF treatment in vivo, we orally administered DMF to 6-month-old *App*^NL−G−F/NL−G−F^ (*App*-KI) and WT mice for 1 month. We isolated microglia and astrocytes from the cerebral cortices by MACS and measured the expression of *Nrf2 (Nfe2l2)* mRNA and its downstream genes using qRT-PCR (Fig. 2A). Intriguingly, we found that the expression of Nrf2-downstream genes was different between microglia and astrocytes after DMF administration. In isolated microglia, only *Gclm* expression was upregulated after DMF treatment (Fig. 2B), whereas *Nqo1* and *Osgin1* expression was upregulated in astrocytes of DMF-administered *App*-KI mice (Fig. 2C). No difference in the expression of *Nfe2l2* was observed between WT and *App*-KI microglia and astrocytes after DMF administration. These results suggest that short-term DMF administration differentially regulates Nrf2-related genes in astrocytes and microglia of *App*-KI mice.

### DMF administration improves cognitive function in *App*-KI mice

**Fig. 3:**
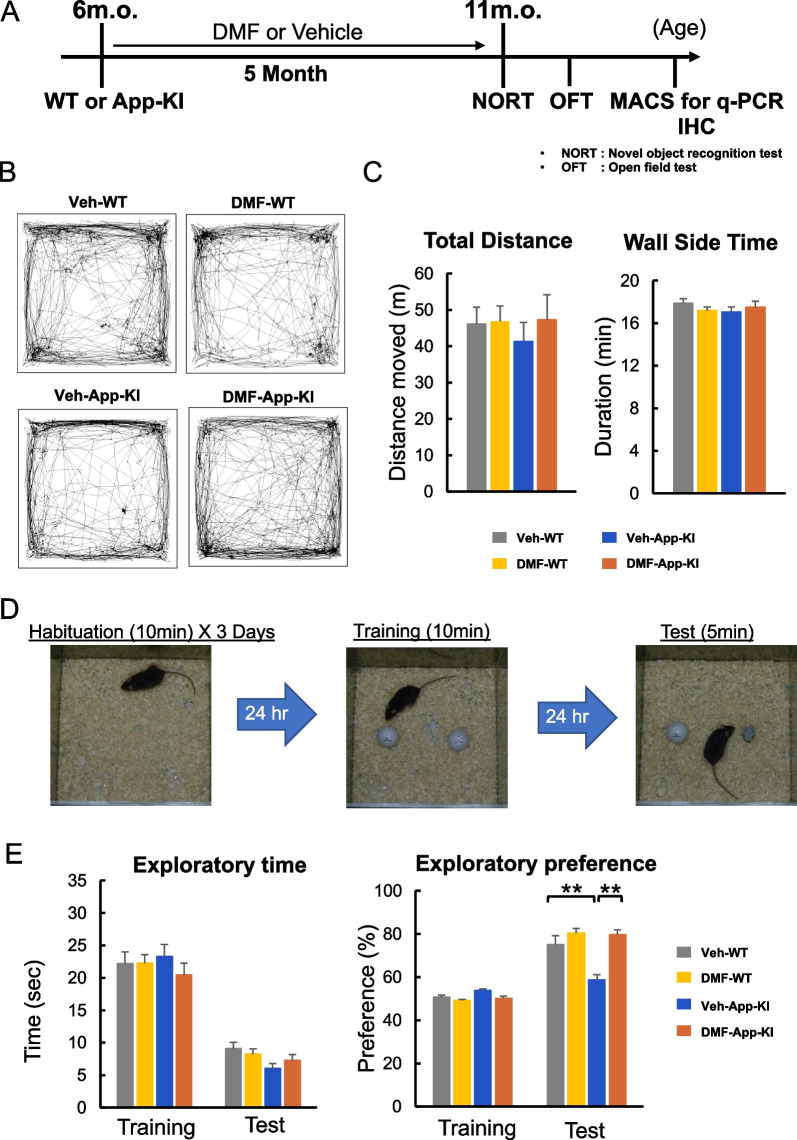
Chronic oral DMF administration improves cognitive dysfunction in *App*-KI mice. **A** Schematic protocol for chronic oral DMF administration and behavioral experiments. Six-month-old WT or *App*-KI mice were orally administered with DMF or vehicle control 3 days per week for 5 months. NORT, novel object recognition test; OFT, open field test; IHC, immunohistochemistry. **B** Representative images showing the travel paths of Veh-WT, DMF-WT, Veh-*App*-KI, and DMF-*App*-KI were recorded for 20 min in the open field test arena. **C** Locomotor activity and anxiety were evaluated in an open field test as the the total distance and wall side time. Values are presented as means ± SEM. [Veh-WT (n = 14), DMF-WT (n = 14), Veh-*App*-KI (n = 7), and DMF-*App*-KI (n = 9)]. *p < 0.05 and **p < 0.01 (two-way ANOVA). **D** An experimental protocol for the novel object recognition test. **E** Performance of the DMF- or vehicle (Veh)-administered WT and *App*-KI mice was assessed using the novel object recognition test at 11 months of age. Values are expressed as means ± SEM [Veh-WT (n = 19), DMF-WT (n = 24), Veh-*App*-KI (n = 19), and DMF-*App*-KI (n = 19)]. *p < 0.05 and **p < 0.01 (two-way ANOVA)

To determine the effects of DMF on the cognitive function of *App*-KI mice, we orally administered DMF to 6-month-old *App*-KI and WT control mice for 5 months. We did not observe significant weight loss, hair loss, or other gross alterations in DMF-administered mice (Additional file 3; Fig. S2). Four groups of mice (vehicle-WT, DMF-WT, vehicle-*App*-KI, and DMF-*App*-KI) were evaluated by behavioral experiments at 11 months of age (Fig. 3A). We first examined whether DMF administration affects spontaneous behavior, such as emotional behavior and locomotor activity, in mice using an open field test. No significant differences in the total distance and wall side time were observed among the four groups (Fig. 3B and C). In the novel object recognition test (Fig. 3D and E), no significant difference in exploration time and preference was observed among the four groups, indicating that DMF did not affect the activity of mice. During the test session, vehicle-administered *App*-KI mice showed significantly less exploratory preference for the novel object than WT mice, as expected. DMF administration significantly improved the preference index in *App*-KI mice (Fig. 3E). These results suggest that although no significant difference in curiosity and motivation for objects or motor function was observed among the four groups, DMF administration significantly improved object recognition performance in *App*-KI mice.

### DMF reduces neuroinflammation in *App*-KI mice

**Fig. 4:**
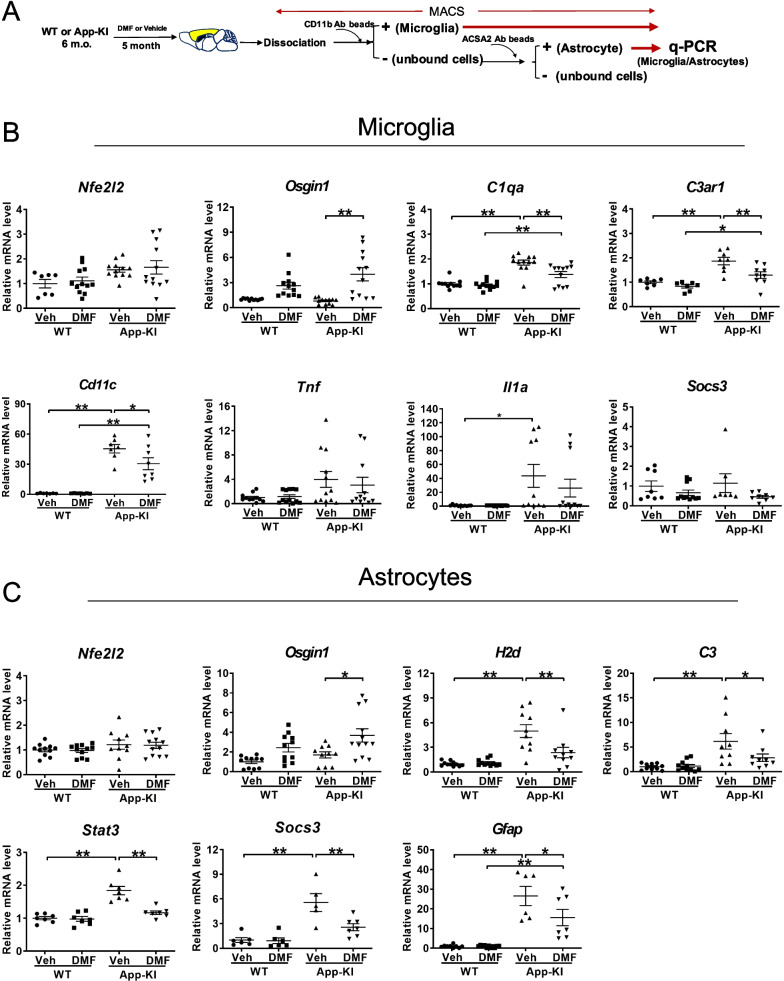
Chronic oral DMF administration suppresses the expression of neuroinflammatory molecules in the glial cells of *App*-KI mice. **A** Schematic overview of gene expression analysis of microglia and astrocytes isolated from the cerebral cortices of DMF- or vehicle-administered WT and *App*-KI mice using MACS. **B** Expression levels of mRNAs in isolated microglia from DMF- or vehicle (Veh) -administered WT and *App*-KI mice determined by quantitative PCR. Relative expression levels of Nrf2 (*Nfe2l2*) and its downstream molecule *Osgin1*, complements and their receptors (*C1qα* and *C3αr*), DAM marker (*Cd11c*), inflammation factors (*Tnf* and *Il1a*), and inflammation regulator (*Socs3*) are plotted as means ± SEM. [Veh-WT (n = 7–12), DMF-WT (n = 6–12), Veh-*App*-KI (n = 7–12), and DMF-*App*-KI (n = 8–12)]. *p < 0.05 and **p < 0.01 (two-way ANOVA). **C** Expression levels of mRNAs in isolated astrocytes from DMF- or vehicle (Veh)-administered WT and App-KI mice determined by quantitative PCR. Relative expression levels of Nrf2 (*Nfe2l2*) and its downstream molecule *Osgin1*, and A1 astrocyte marker, (*H2d*), a complement (*C3*), inflammation regulators (*Socs3* and *Stat3*), and astroglial activation marker (*Gfap*) are represented as means ± SEM. [Veh-WT (n = 6–11), DMF-WT (n = 6–11), Veh-App-KI (n = 5–9), and DMF-App-KI (n = 7–12)]. *p < 0.05 and **p < 0.01 (two-way ANOVA)

To further investigate the molecular basis of the beneficial role of DMF in *App*-KI mice, we sequentially isolated Cd11b^+^ microglia and ACSA2^+^ astrocytes from the cerebral cortices of 11-month-old mice using MACS and then examined the gene expression in glial cells using qRT-PCR (Fig. 4A). As expected, we found that *Osgin1,* an Nrf2 target gene, was upregulated in microglia and astrocytes of DMF-administered *App*-KI mice, whereas the levels of *Nfe2l2* were not altered (Fig. 4B, C). This is consistent with the fact that DMF activates Nrf2 through posttranslational regulation, such as the dissociation of Keap1 from Nrf2. Furthermore, we found that DMF administration downregulated the expression of the disease-associated microglia (DAM) marker *Cd11c*, complement *C1qa,* and its receptor *C3ar1* in *App*-KI microglia (Fig. 4B). We then examined the expression levels of reactive astrocyte markers and found that the levels of *H2d* and *C3,* which were upregulated in *App*-KI astrocytes, were suppressed by DMF administration (Fig. 4C). Activated microglia are known to secrete IL-1α, TNFα, and C1q to induce A1 reactive astrocytes in vitro [34]. Therefore, we measured the expression of *Il1a*, *Tnf*, and *C1qa* in isolated microglia, and found that *C1qa* expression in *App*-KI microglia was suppressed by DMF (Fig. 4B).

A recent study showed that STAT3, a direct target of C3-C3aR signaling, functionally mediates tau pathology [41]. Therefore, to determine whether Nrf2 activation regulates neuroinflammation in astrocytes, we examined the expression levels of *Stat3* and *Socs3*, inflammatory regulators, and *Gfap*, a representative marker for reactive astrocytes. The expression levels of *Stat3*, *Socs3*, and *Gfap* were significantly downregulated in the astrocytes of DMF-administered *App*-KI mice (Fig. 4C). These results suggest that DMF-mediated Nrf2 activation inhibits reactive astrocyte markers and suppresses the astrocytic STAT3 and SOCS3 signaling pathways to improve neuroinflammation in *App*-KI mice.

### DMF administration reduces dystrophic neurites without altering amyloid-β clearance in *App*-KI mice

**Fig. 5:**
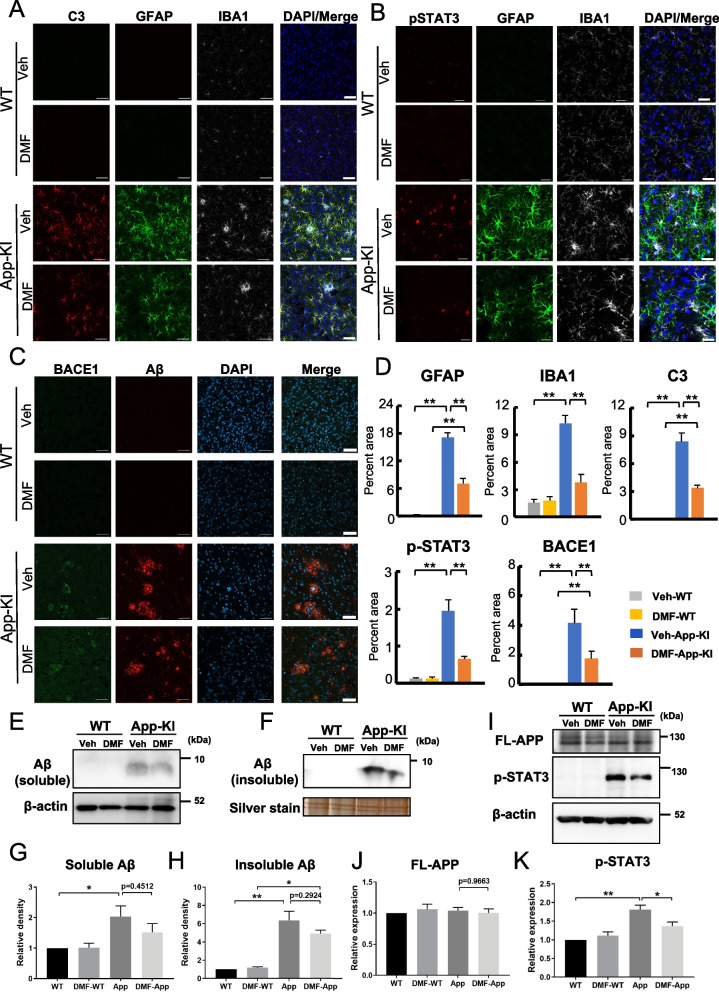
Neuroinflammation reduced in the cerebral cortices of DMF-administered *App*-KI mice. **A**–**C** Representative immunofluorescent images of C3 (red), GFAP (green), and IBA1 (gray) expression (**A**); pSTAT3 (red), GFAP (green), and IBA1 (gray) expression (**B**); and BACE1 (green) and Aβ (red) expression (**C**) in the cerebral cortices of DMF- or Veh-administered WT and *App*-KI mice at 11 months of age. DAPI and merged images are also shown (**A**–**C**). Scale bars: 50 μm (**A**–**C**). **D** Quantification of the immunofluorescence images in **A**–**C**. Percentage of areas immunopositive for GFAP, C3, IBA1, pSTAT3, and BACE1 in the cerebral cortices of Veh- or DMF-administered WT (gray, yellow) and *App*-KI mice (blue, orange). Values are presented as means ± SEM. [n = 3. Three slices per mouse were quantified]. **p < 0.01 (two-way ANOVA). **E**–**H** Representative immunoblotting images for soluble Aβ level in TBS-soluble fractions (**E**) and insoluble Aβ level in formic acid (FA)-soluble fractions (**F**) extracted from the cerebral cortices of Veh / DMF-administered WT and *App*-KI mice at 11 months of age. The immunoblots for β-actin (**E**) and silver staining of SDS-PAGE (**F**) are shown as loading controls. For the TBS-soluble fractions (**E**) and FA-soluble fractions (**F**), 30 and 2 µg of the protein were loaded per well, respectively. The relative levels of soluble and insoluble Aβ were quantified from four independent experiments and are expressed as means ± SEM with *p*-values (**G**, **H**, n = 3). **I**–**K** Representative immunoblotting images for full-length APP (FL-APP) and pSTAT3 levels in the cerebral cortices of Veh/DMF-administered WT and *App*-KI mice at 11 months of age (**I**). Five µg of the protein was loaded per well (**I**). The levels of FL-APP and pSTAT3 were quantified and are expressed as means ± SEM (**J**, **K**, n = 3). *p < 0.05 and **p < 0.01, two-way ANOVA (**G, H, J, K**)

To confirm that DMF reduces neuroinflammation in *App*-KI mice, we performed immunofluorescence analysis of the cerebral cortices of DMF- or vehicle-administered WT and *App*-KI mice. The expression of GFAP, C3, and IBA1 was substantially increased in the cerebral cortices of *App*-KI mice. Chronic administration of DMF significantly inhibited the expression of GFAP and IBA1 in *App*-KI mice (Fig. 5A, B, and D and Additional file 3: Fig. S3). Furthermore, the immunoreactivity of C3 and pSTAT3 mainly overlapped with GFAP-positive astrocytes in *App*-KI mice and was substantially reduced with DMF administration (Fig. 5A, B, and D). In contrast, no significant difference in Aβ immunoreactivity was observed between *App*-KI mice with and without DMF administration (Fig. 5C, D). Because BACE1 accumulation represents dystrophic presynaptic terminals [42], we examined the expression of BACE1 in WT and *App*-KI mice. DMF administration reduced BACE1-positive dystrophic nerve terminals in *App*-KI mice (Fig. 5C, D). The immunofluorescence results of Aβ were also confirmed by immunoblotting of soluble and insoluble Aβ (Fig. 5E–H) and full-length APP (Fig. 5I, J), suggesting that DMF-mediated Nrf2 activation does not affect Aβ production and clearance. The protein level of p-STAT3 was significantly decreased in DMF-administered App-KI mice, indicating that oral administration of DMF inhibited the STAT3 pathway (Fig. 5I, K). These results indicate that chronic DMF administration reduces neuroinflammation and dystrophic neurites in *App*-KI mice likely through activating the Nrf2 pathway.

## Discussion

In the present study, we found that chronic oral administration of DMF reduced neuroinflammation, particularly in astrocytes, and reversed cognitive dysfunction in *App*-KI mice. Moreover, detailed gene expression analysis of MACS-isolated cortical microglia and astrocytes revealed that DMF inhibited the C3-STAT3 pathway, presumably through microglia–astrocyte interaction, in *App-*KI mice.

Chronic oral DMF administration conferred neuroprotection in *App*-KI mice with improved performance in the novel object recognition test and reduced dystrophic neurites. Furthermore, studies have investigated the therapeutic potential of DMF in various AD and dementia models. Rojo and colleagues showed that DMF reduced the expression of glial markers and reversed cognitive impairment in APP^V717I^ and Tau^P301L^ double transgenic mice [10]. El-Fatah et al. showed that DMF improved cognitive deficits and reduced neuroinflammation in d-galactose-treated ovariectomized (OVX) rats in a postmenopausal dementia model [43]. In contrast, Mohle and colleagues reported that DMF did not reduce cognitive decline and amyloid-β deposition in female APP/PS1 mice [26, 44]. Differences in the results among these studies, including ours, may be attributed to the use of different mouse models, the protocol of DMF administration (dose and duration), and the methods used for assessing cognitive function. Compared with other cited studies, our gene expression analysis of MACS-separated microglia and astrocytes revealed that DMF attenuated the expression of proinflammatory genes, particularly in astrocytes of *App*-KI mice, suggesting that activation of the Nrf2 pathway inhibits inflammatory reactive astrocytes through microglia–astrocyte interaction.

Neuroprotection mediated by the Nrf2-ARE pathway has been well described in astrocytes, probably because the Nrf2 target genes conferring protection against oxidative stress are enriched in astrocytes [45]. Furthermore, our gene expression analysis also revealed that the expression of Nrf2 target genes, *Hmox1, Gclm, Nqo1,* and *Osgin1,* was increased in cortical astrocytes isolated from DMF-administered *App*-KI mice (Fig. 2). Although previous studies have demonstrated anti-inflammatory effects of DMF in astrocytes via the Nrf2 pathway [39, 40], few studies have focused on the role of DMF in proinflammatory A1 astrocytes. Our data suggest that DMF administration inhibits inflammatory reactive astrocytes. Microglia may contribute to the induction of such astrocytes, also known as A1 astrocytes [34]. Complement component C3 is one of the most characteristic and highly upregulated molecules in A1 astrocytes [34]. C3 is primarily expressed in astrocytes, where its expression is upregulated in *App*-KI mice, and DMF inhibited C3 expression in astrocytes. C3aR, the receptor for C3a, is highly expressed in microglia and its expression is particularly prominent in the areas surrounding amyloid plaques in APP transgenic mice [46]. In mice with AD, increased interaction of activated astrocyte-derived C3 with microglial C3aR may impair microglial phagocytosis and thus microglial Aβ clearance [46]. In this study, we found that the expression of *C3* and *C3ar* was significantly suppressed in astrocytes and microglia isolated from DMF-administrated *App*-KI mice, respectively. These results, including those of the related studies cited above, suggest that astrocyte–microglia interaction contributes to complement inhibition in AD mice.

In this study, DMF had a beneficial effect on C3 expression and, through C3-mediated STAT3 signaling, inhibited *Stat3* and its downstream gene *Socs3* in mice with AD. STAT3 signaling has been reported as a critical regulator of reactive astrogliosis in the disease states including spinal cord injury [47]. In AD models, inhibition of STAT3-mediated astrogliosis or its phosphorylation reduces pathological changes in the brains of APP/PS1 and 5XFAD mice [48, 49]. Reducing STAT3 signaling by inhibiting C3–C3aR signaling alleviates neuroinflammation in a tau model of AD [41]. Consistent with these observations, our results suggest that suppression of the C3-mediated STAT3 pathway is important for controlling the pathogenesis of AD [50].

This study has some limitations. Neuronal Nrf2 signaling was not examined because of the inability to isolate neurons from adult mouse brains using MACS. Although Nrf2 targets are enriched in astrocytes, DMF may provide protection through neuronal Nrf2 signaling. Furthermore, C3aR is also expressed in neurons [16, 51], suggesting that C3–C3aR signaling is mediated by neuron–astrocyte interaction. The neuronal contribution of DMF-mediated neuroprotection in AD models will be investigated in future studies. Another limitation is that the sex-specific effect of DMF was not determined. There is an intriguing report that DMF decreased microglia activation only in aged female mice [52]. Differences in the transcriptome and phenotype of microglia in male and female brains have been well described [53, 54]. Because of the limited sample size, our study could not address the sex-specific features of neuroinflammation in DMF-administered mice with AD. Therefore, there is room for further investigation of the sex-specific features of the neuroinflammatory phenotype in DMF-administered mice with AD.

In conclusion, our study identified the activation of astrocytic Nrf2 signaling as a viable therapeutic target in AD by controlling neuroinflammation, particularly through the regulation of C3-STAT signaling in astrocytes. Furthermore, it raised the possibility of repositioning DMF as a drug for treating neurodegenerative diseases, including AD.

### Supplementary Information

**Additional file 1.** A list of the primers used for this study.

**Additional file 2.** Summary of statistical analysis.

**Additional file 3.** Supplementary figures.

## Data Availability

All data generated or analyzed during this study are included in this published article.

## Abbreviations

Aβ: Amyloid β
AD: Alzheimer’s disease
APP: Amyloid precursor protein
C1q: Complement component 1, q subcomponent
C3: Complement component 3
C3aR: Complement component 3a receptor
Cd11c (Itgax): Integrin subunit alpha X
DAM: Disease-associated microglia
DMF: Dimethyl fumarate
Gbp2: Guanylate binding protein 2
Gclm: Glutamate-cysteine ligase modifier subunit
H2d: Histocompatibility 2, D region
H2t23: Histocompatibility 2, T region locus 23
Homx1: Heme oxygenase 1
Keap1: E3 ligase adapter Kelch-like ECH-associated protein 1
Nrf2: Nuclear factor-erythroid 2-related factor 2
Osgin1: Oxidative stress induced growth inhibitor 1
SOCS3: Suppressor of cytokine signaling 3
STAT3: Signal transducer and activator of transcription 3
