# Supplementary material for: Dimethyl fumarate improves cognitive impairment and neuroinflammation in mice with Alzheimer’s disease
Source: J Neuroinflammation. 2024 Feb 21;21:55. doi: 10.1186/s12974-024-03046-2 (PMC10882778; doi:10.1186/s12974-024-03046-2)
Supplement: Supplementary file 1 — Additional file 1. A list of the primers used for this study. [file 12974_2024_3046_MOESM1_ESM.pdf]

Table S1. A list of the primers used for this study

Transcript	Forward	Reverse
Hmox1	5′-GGACCCATGTCTTCGGAAAT-3’	5’-CCCATGACACCTCTCTCCAT-3'
Gclm	5’- GCCACCAGATTTGACTGCCTTTG-3’	5’- TGCTCTTCACGATGACCGAGTACC-3'
H2-d	5′-CATTGACCGCTACCTGAAGACC-3’	5′-GCCTCCTGTTGGTGAGAATCATG-3'
H2-t23	5′-TGTCAAGTTCCCAGAAATGTTCCA-3’	5′-ATGCCGCCGTTCTGAATGA-3'
Gbp2	5′-GAACCGCTTCTGGGATTACCTG-3’	5′- GCCTTTACTTCCGTCATAGTGTC-3'
C3	5'-CCAGCTCCCCATTAGCTCTG-3'	5'-GCACTTGCCTCTTTAGGAAGTC-3'
Socs3	5′-TACTGAGCCGACCTCTCTC-3’	5′-AGCTGGGTCACTTTCTCATA-3'
Gfap	5′-ACCAGTGGAGAATCCCCTATAC-3’	5′-GCCAAGACTGTAGTGTGGTCA-3’
Nfe2l2	5′-CTGGAACGGTGAAGGTGACA-3′	5′-CGGCCACATTGTGAACTTTG-3'
Nqo1	5’- GCGAGAAGAGCCCTGATTGTACTG-3'	5’- TCTCAAACCAGCCTTTCAGAATGG-3'
Osgin1	5'-CGGTGACATCGCCCACTAC-3'	5'-GCTCGGACTTAGCCCACTC -3'
C1qα	5'-CTCAGGGATGGCTGGTGGCC-3'	5'-CCTTTGAGACCCGGCCTCCCC-3'
C3ar	5′-ACCAGTGGAGAATCCCCTATAC-3’	5′-GCCAAGACTGTAGTGTGGTCA-3’
Cd11c	5’-TCGTATTTGGCTTCCCAGAC-3’	5’-CCATCATTAGACACCGTCACAT-3’
Tnf	5’-CCCAAGGCGCCAGATC-3’	5’-TCGCGGATCATGCTTTCTG-3’
Il1α	5’-CGCTTGAGTCGGCAAAGAAAT-3’	5'-CTTCCCGTTGCTTGACGTTG-3'
Stat3	5′-TAGCCGATTCCTGCAAGAGTCCAA-3’	5′-CGGGCAATTTCCATTGGCTTCTCA-3’
Actb	5′-ACCAGTGGAGAATCCCCTATAC-3’	5′-GCCAAGACTGTAGTGTGGTCA-3’
