# Supplementary material for: Dimethyl fumarate improves cognitive impairment and neuroinflammation in mice with Alzheimer’s disease
Source: J Neuroinflammation. 2024 Feb 21;21:55. doi: 10.1186/s12974-024-03046-2 (PMC10882778; doi:10.1186/s12974-024-03046-2)
Supplement: Supplementary file 3 — Additional file 3. Supplementary figures. [file 12974_2024_3046_MOESM3_ESM.pdf]

Additional file 3 (Supplementary figures)

Dimethyl fumarate improves cognitive impairment and neuroinflammation in mice with Alzheimer's disease

Ting Wang, Akira Sobue, Seiji Watanabe, Okiru Komine, Takaomi C Saido, Takashi Saito, and Koji Yamanaka

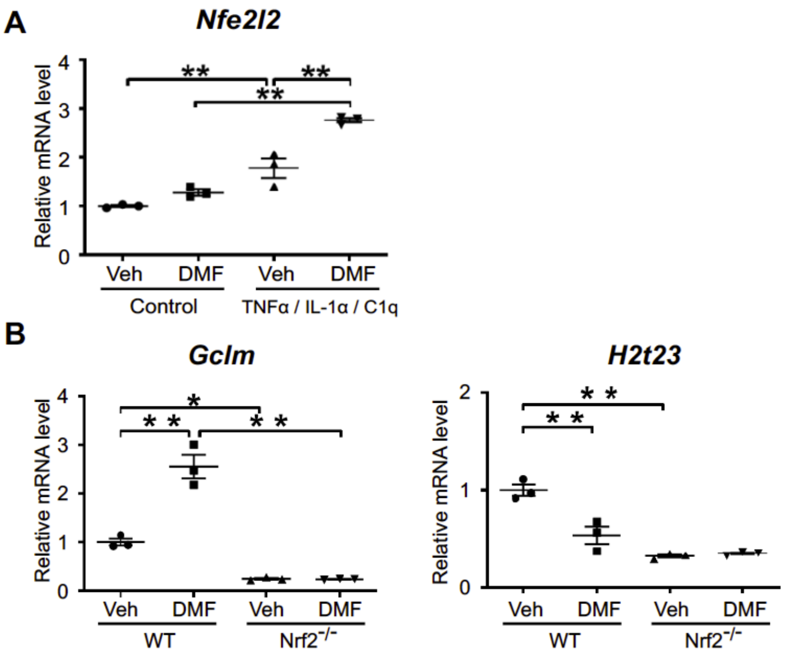

Figure S1. Dimethyl Fumarate (DMF) activates the Nrf2 pathway and inhibits proinflammatory activation in primary astrocytes

A. Expression levels of mRNAs in DMF-treated primary astrocytes determined by quantitative PCR. Relative expression levels for *Nfe2l2* are plotted as means ± SEM (Veh-Control: n = 3, DMF-Control: n = 3, Veh-A1 (TNFα/IL-1α/C1q): n = 3, and DMF-A1 (TNFα/IL-1α/C1q): n = 3). $^{**}p$ <0.01, two-way ANOVA.

B. Expression levels of mRNAs in A1-induced primary astrocytes derived from WT and $Nrf2^{-/-}$ mice determined by quantitative PCR. Relative expression levels for *Gclm* and *H2t23* are plotted as means ± SEM (Veh-WT: n = 3, DMF-WT: n = 3, Veh-$Nrf2^{-/-}$: n = 3, and DMF-$Nrf2^{-/-}$: n = 3). $^{*}p$ <0.05 and $^{**}p$ <0.01, two-way ANOVA.

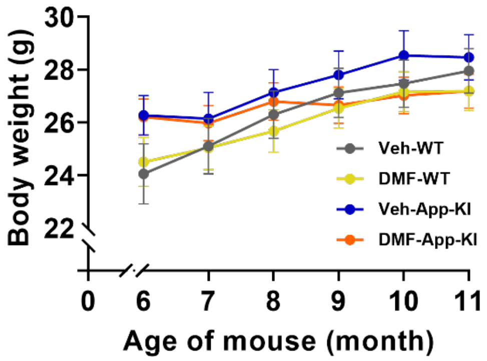

Figure S2. No difference in the body weight among each group.

The body weights were quantified and are expressed as means ± SEM [Veh-WT (n = 19), DMF-WT (n = 24), Veh-*App*-KI (n = 19), and DMF-*App*-KI (n = 19)]. No statistical significance was found. Repeated measure two-way ANOVA.

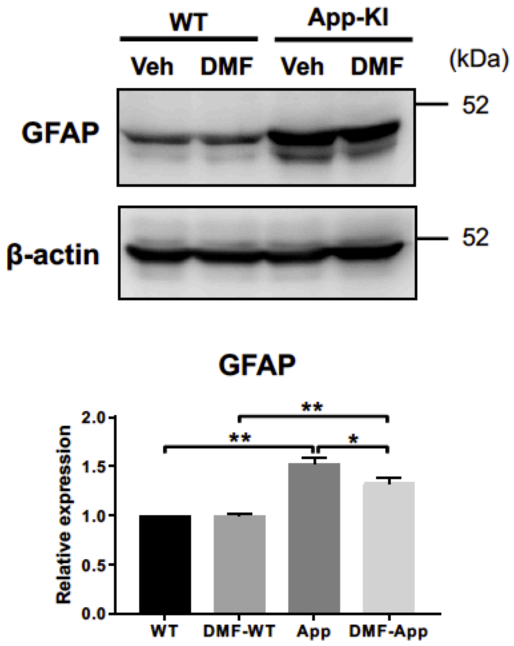

Figure S3. Glial fibrillary acidic protein suppressed in the cerebral cortices of DMF-administered App-KI mice

Representative immunoblotting images for GFAP levels in the cerebral cortices of Veh / DMF administered- WT and App-KI mice at 11 months old. Five μg of the protein was loaded per lane. The GFAP levels were quantified and are expressed as means ± SEM (J, n=3). *p <0.05 and **p <0.01, two-way ANOVA.
